# Biosurfactant-driven desorption and remediation of heavy oil contaminated soils underpinned by molecular simulations and microbial dynamics

**DOI:** 10.1039/d5ra09479h

**Published:** 2026-03-25

**Authors:** Qi Xiu, Honglin He, Zhenghui Liu, Xuan Ou, Yifei Meng, Kangbo Zhao, Qian Yang, Xinrui Zhang, Yahan Hou, Shun Yao, Peike Gao, Wenjie Xia

**Affiliations:** a Key Laboratory of Molecular Microbiology and Technology, Ministry of Education, College of Life Sciences, Nankai University Tianjin 300071 P. R. China wenjie.xia@nankai.edu.cn; b Tianjin Academy of Agricultural Sciences Huada Road, 17th Kilometric Marker of Jinjing Highway, Xiqing District Tianjin 300381 China; c College of Life Sciences, Qufu Normal University Qufu Shandong 273165 P. R. China

## Abstract

This study integrates molecular dynamics simulations and bench-scale experiments to investigate the adsorption and desorption behaviors of heavy oil on five mineral substrates: SiO_2_, kaolinite, muscovite, and Ca^2+^-/Na^+^-montmorillonite. Adsorption followed Langmuir isotherms, with montmorillonite exhibiting the highest capacities (0.061–0.062 molecules per Å^2^ for aromatics in simulations; 0.086–0.091 g g^−1^ in bench-scale tests) and SiO_2_ the lowest (0.027 pcs per Å^2^; 0.013 g g^−1^). Among four biosurfactants evaluated—rhamnolipid, sophorolipid, trehalose lipid, and mannosylerythritol lipid–sophorolipid consistently achieved the greatest desorption efficiency, removing up to 99.63% of adsorbed oil from Na^+^-montmorillonite and 96.04% from field-contaminated soil. 16S rRNA and metagenomic sequencing revealed an increased abundance of hydrocarbon-degrading bacteria within the soil microbial community, highlighting a synergistic effect between biosurfactant-induced desorption and biodegradation. These findings underscore the critical roles of mineralogical properties, oil fraction characteristics, and biosurfactant selection in soil washing treatment. This work presents a viable and eco-friendly strategy for remediating crude oil-contaminated soils, with important implications for optimizing large-scale environmental restoration efforts.

## Abstract 2

This study integrates molecular dynamics simulations and bench-scale experiments to investigate the adsorption and desorption behaviors of heavy oil on five mineral substrates: SiO_2_, kaolinite, muscovite, and Ca^2+^-/Na^+^-montmorillonite.

## Graphical abstract

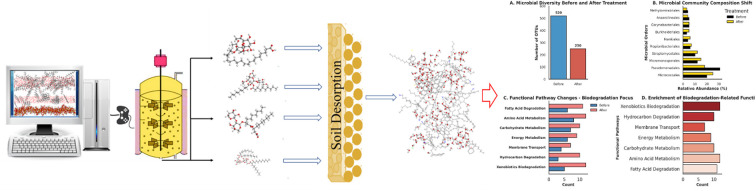

## 1. Introduction

Crude oil contamination in soil poses a significant global environmental challenge, particularly in oilfield regions where spills occur frequently and can persist for extended periods.^1,2^ Petroleum hydrocarbons, especially complex fractions such as asphaltenes and resins, are notably persistent and can degrade soil quality for decades.^3^ Even minor oil leaks can contaminate large areas-one liter of oil can affect up to 3784 m^2^ of soil-leading to diminished agricultural productivity and posing ecological risks.^4,5^ At sufficiently high contamination levels (>1 g kg^−1^), crude oil interferes with plant root development and reduces germination rates of staple crops by nearly half, with adverse impacts also documented for soil fauna.^6,7^

Various remediation strategies have been employed to address such contamination, including chemical leaching, solvent extraction, phytoremediation, thermal treatment, and ultrasonic methods.^8,9^ However, these techniques often face practical limitations-chemical leaching, for instance, can be inefficient for low-concentration pollutants and may generate secondary pollution through sludge and wastewater production.^10,11^ Consequently, there is a growing demand for innovative, eco-friendly remediation approaches that minimize environmental damage while effectively removing residual hydrocarbons.

A key step in optimizing such strategies is developing a clear understanding of pollutant–mineral interactions. Soil mineralogy—particularly the presence of swelling clays like montmorillonite or non-expanding clays such as kaolinite, significantly affects adsorption and desorption processes.^12,13^ High cation exchange capacity (CEC) and larger interlayer spacing can enhance the affinity of soil minerals for various hydrocarbon fractions.^14^ Moreover, the diverse chemical composition of crude oil, including saturates, aromatics, resins, and asphaltenes, influences the strength with which these pollutants bind to mineral surfaces.^15–17^ Soil mineral composition, comprising primary and secondary clay minerals, directly influences contamination processes and remediation efficacy. Dominant minerals such as montmorillonite (a 2 : 1 clay with high CEC) and kaolinite (a 1 : 1 clay with moderate CEC) act as key sorbents for heavy metals (*e.g.*, Pb^2+^ and Cd^2+^) *via* electrostatic attraction and surface complexation, while quartz (SiO_2_) primarily affects contaminant mobility through its textural control on soil pore water.^18^ Recent studies highlight that clay-rich soils with elevated montmorillonite content exhibit enhanced retention of organic pollutants (such as polycyclic aromatic hydrocarbons (PAHs)) owing to their large specific surface area and hydrophobic interactions.^19^ Conversely, kaolinite-dominated soils may require augmented remediation strategies to mitigate contaminant leaching, as their lower CEC limits natural sorption capacity.^20^ These complexities underscore why an integrative approach—combining computational and experimental techniques, is necessary for developing targeted remediation solutions. The selection of the five minerals examined in this study is therefore justified by their critical roles in soil pollution control.

Recently, biosurfactants have emerged as promising alternatives for soil washing due to their biodegradability, low toxicity, and reduced risk of secondary pollution compared to synthetic surfactants, consequently, they have been widely applied. Existing biosurfactant-based soil remediation techniques have primarily focus on single-performance evaluation and empirical screening. Most studies have verified the desorption or biodegradation efficiency of one or several biosurfactants on contaminated soil through bench-scale experiments, and explored the influence of environmental factors such as pH, temperature and soil–water ratio on remediation effect. Some studies have combined biosurfactants with microbial agents or physical methods to form integrated remediation strategies, which have improved remediation efficiency to some extent. However, these existing studies still exhibit notable limitations: first, the screening of biosurfactants mostly relies on a large number of repeated experimental tests, which is time-consuming, labor-intensive, and inefficient, and it is difficult to realize the targeted selection of biosurfactants for different contaminated soil types; second, the research on the remediation mechanisms of biosurfactants is mostly limited to the macroscopic level, and the molecular interaction mechanism between biosurfactants, petroleum hydrocarbons and soil mineral surfaces has not been deeply elucidated; third, most studies focus on a single aspect—either desorption or biodegradation, and lack the systematic research on the synergistic effect between biosurfactant-induced desorption and indigenous microbial biodegradation in soil. Furthermore, the influence of soil mineral composition—a core factor affecting the adsorption and desorption of petroleum hydrocarbons—on the remediation efficiency of biosurfactants is often ignored or only superficially discussed in existing studies, this gap leads to the limited applicability of the developed remediation strategies in actual heterogeneous contaminated soils.

In this study, we aimed to develop a high-efficiency and rapid screening protocol for soil desorbents using molecular dynamics simulations. This approach seeks to circumvent the conventional large-scale experimental screening workflow, thereby significantly enhancing screening efficiency and target specificity, reducing screening costs, and—critically—providing comprehensive elucidation of the desorption mechanisms of high-performance desorbents. To this end, we investigated the adsorption and desorption mechanisms of various components of heavy oil on common minerals (SiO_2_, kaolinite, muscovite, and Ca^2+^-/Na^+^-montmorillonite). Additionally, we calculated the desorption efficiencies of several novel biosurfactants, including rhamnolipid (RL), sophorolipid (SL), trehalose lipid (TL), and mannosylerythritol lipid (MEL). Rhamnolipids (from *Pseudomonas* spp.) and sophorolipids (from *Candida* spp.) are the most industrially mature biosurfactants, widely applied in petroleum bioremediation and cosmetics. Meanwhile, mannosylerythritol lipids (MELs) and trehalose lipids, —produced by fungi—have also scaled up commercially for oil recovery and eco-detergent uses.^21,22^ Recently, a study combining molecular dynamics simulations with experimental synthesis has further demonstrated the targeted desorption efficiency of structure-optimized MELs for specific pollutants, validating their potential in precision soil remediation.^23^ However, the industrial application of other biosurfactant types still requires substantial development. Laboratory and field validations confirmed the accuracy of this workflow and model. This research provides a comprehensive framework for designing high-efficiency, environmentally friendly desorbents tailored to the mineralogical and contaminant characteristics of contaminated soils.

## 2. Materials and methods

### 2.1 Samples

Contaminated soil samples were collected from a wellhead in the Jilin Oilfield, which has experienced chronic petroleum pollution for over five years. Surface soil (0–20 cm) was sampled using a stainless-steel shovel to minimize cross-contamination. Approximately 3–5 subsamples were combined into a composite sample of approximately 5 kg, placed into airtight polyethylene bags, and stored at 4 °C until analysis. Crude oil samples were also obtained from the same site to maintain consistency between contamination sources. The composition of the crude oil (saturates, aromatics, resins and asphaltenes; SARA) was determined following the methodology described by Rosa N. M. A.^24^ The procedure begins by dissolving the insoluble asphaltene fraction with *n*-pentane. Subsequently, is used to elute the remaining components sequentially with *n*-pentane and toluene. The components retained within the chromatographic system are classified as resin fraction. Each fraction is then analyzed separately for its content. Standard mineral samples, including Ca^2+^-montmorillonite, kaolinite, muscovite, Na^+^-montmorillonite, and SiO_2_, were procured from a certified supplier (*e.g.*, the Clay Minerals Society). All mineral samples had a purity of ≥99%. The BET surface areas were as follows: ≥80 m^2^ g^−1^ for both types of montmorillonite, ≥3.5 m^2^ g^−1^ for muscovite, and ≥20 m^2^ g^−1^ for kaolinite. All mineral samples were dried at 80 °C for 2 h prior to use.

### 2.2 Analysis of soil samples in the field

Field-collected soil samples were further characterized to inform the selection of an optimal desorbent. X-ray diffraction (XRD) analyse were performed using a Rigaku D/max-2500 diffractometer (Rigaku, Japan) equipped with a Cu Kα radiation source operated at 40 kV and 200 mA. A graphite monochromator filter was used to reduce background noise. Each sample was ground to pass through a 100-mesh sieve, mounted on a glass slide, and scanned over a 2*θ* range of 5–60° in increments of 0.02°. Diffraction peaks were matched against the ICDD PDF-2 database, and Rietveld refinement was employed for semi-quantitative determination of mineral phases. This resulting compositional data guided the selection and optimization of biosurfactant-based treatments.

### 2.3 Adsorption and desorption simulations

#### 2.3.1 Model setup

Molecular dynamics (MD) simulations were conducted using Materials Studio 2019 (BIOVIA, USA) to elucidate the adsorption and desorption processes of petroleum hydrocarbons on various mineral surfaces, both in the presence and absence of biosurfactants. The minerals studied included Na^+^-montmorillonite (Na_0.75_^+^(Si_7.75_Al_0.25_)(Al_3.5_Mg_0.5_)O_20_(OH)_4_ (CEC = 108 mmol kg^−1^)), muscovite (K [AlSi_3_O_8_][Al_2_O_22_(OH)_2_] (CEC = 25 mmol kg^−1^)), kaolinite ([SiO_4_][Al_2_O(OH)_4_] (CEC = 0 mmol kg^−1^), Ca^2+^-montmorillonite: Ca_0_._325_^2+^(Si_7.75_Al_0.25_)(Al_3.5_Mg_0.5_)O_20_(OH)_4_ (CEC = 256 mmol kg^−1^)), and SiO_2_(quartz). The annealing relaxation temperature was rigorously controlled within the range of 298 K to 800 K. During the annealing phase, two main types of surface defects were observed: oxygen atoms bonded to only one silicon atom, resulting in incomplete coordination, and silicon atoms lacking full coordination. To address these structural imperfections arising from atomic unpairing during relaxation, –OH and –H groups were attached to the cleaved surface, yielding in a fully hydroxylated model of the mineral surface.

Four biosurfactants, namely rhamnolipid (RL), sophorolipid (SL), trehalose lipid (TL), and mannosylerythritol lipid (MEL)—were incorporated into the simulations, with molecular structures defined as follows: RL (Rha-Rha-C_12_), SL (Lactonic, di-acetylated sophorolipid), TL (Trehalose mycolate,TMM), MEL (MEL-B/C with a C_12_ fatty acid chain).

#### 2.3.2 Simulation procedure

Each mineral substrate was cleaved along the (100) plane, yielding a slab thickness of approximately 5 Å vacuum gap along the *z*-axis. The simulation cell measured 8.94 × 14.80 × 10.36 Å^3^ and contained 110 substrate atoms. Biosurfactant systems were constructed at a concentration of 1%, consisting of 2 biosurfactant molecules and 98 water molecules per simulation cell.

A representative crude oil model, reflecting the SARA proportions determined experimentally, was constructed using 100 hydrocarbon molecules and was placed approximately 5 Å above the mineral surface. Pre-equilibration was performed for 200 ps under the NVT ensemble (constant number of particles, volume, and temperature) at 298 K to facilitate initial adsorption of oil onto the substrate. Subsequently, a vacuum box containing the biosurfactants was positioned 5 Å above the adsorbed layer, and an additional 10 Å vacuum space was introduced to minimize boundary effects. The full system was simulated for 5 ns under NVT conditions at 298 K, using the COMPASS force field, with a van der Waals cutoff distance of 15.5 Å, and a force field precision of 1.0 × 10^−6^. Mineral atoms were fixed throughout all simulations to isolate the effects of adsorbates and biosurfactants on adsorption and desorption dynamics.

#### 2.3.3 Adsorption isotherms and remediation indices

Adsorption isotherms for crude oil components on the four minerals were computed using the Sorption module with the Metropolis method, covering partial pressures from 10 to 1000 kPa at temperatures of 288, 298, and 308 K. This approach enabled investigation of the effects of mineral composition, temperature, and moisture content on both adsorption and subsequent remediation efficiency. Remediation performance was assessed by evaluating the following parameters:

Interaction energy between pollutants and mineral substrates, calculated according to the method described by Bin^25^ and Olasanmi.^26^*E*_adsorption_ = *E*_total_ − (*E*_surface_ + *E*_contaminants_) − *E*_H_2_O_.

*E*_total_ refers to the total energy of the system at the end of the simulation, *E*_surface_ refers to the energy of interaction between the substrate and the surface, *E*_contaminants_ refers to the energy of interaction between the surface and the contaminant, and *E*_H_2_O_ refers to the sum of internal energies possessed by the water molecules in the system.

Hydrogen bond formation between crude oil components and biosurfactants, indicating potential emulsification or micelle formation.

Mean square displacement (MSD) of pollutant molecules, reflecting their mobility under varying surface conditions.

### 2.4 Bench-scale validation using standard minerals

To verify the findings from the MD simulations, bench-scale desorption experiments were conducted using natural mineral samples pre-saturated with crude oil. Specifically, 2 g of each air-dried, oil-saturated mineral were placed in 50 mL centrifuge tubes, followed by the addition of 2 mL of 1% biosurfactant solution (resulting in a water-to-solid ratio of 1 : 1). The mixtures were shaken at 200 rpm for 2 h at 25 °C.^27^ After incubation the supernatant was separated by centrifugation (5000×*g*, 10 min), and the crude oil content in the liquid phase was quantified by gas chromatography-flame ionization detection (GC-FID). Desorption efficiency was calculated based on the difference in peak areas before and after washing. GC-FID analysis was performed using a non-polar capillary column (*e.g.*, DB-5, 30 m × 0.32 mm × 0.25 µm), with nitrogen as carrier gas, the temperature program was as follows: 50 °C held for 5 min, increased to 300 °C at a rate of 5 °C min^−1^, and held at 300 °C for 10 min, the injection port and detector temperatures were set at 250 °C and 300 °C, respectively.
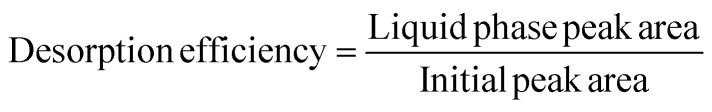


A 1 : 1 water-to-soil ratio is increasingly recognized as optimal for enhancing contaminant removal efficiency in soil remediation, balancing extraction efficacy with resource use. Recent studies have demonstrated that this ratio effectively mobilizes heavy metals and organic pollutants through enhanced washing or microbial degradation, while minimizing excess water input.^28,29^

### 2.5 Bench-scale validation using oilfield soil

Following the simulations and bench-scale tests on standard minerals, the most effective biosurfactant formulation was selected for further validation using field-contaminated soil. The soil was first subjected to impurity removal, then dried at 80 °C for 2 h, crushed, and sieved to pass through a 100-mesh sieve (150 µm). This sieving step was employed to standardize particle size, as soil particle size is known to significantly influence adsorption and desorption processes. Smaller particles provide a larger specific surface area for contaminant adsorption, whereas larger particles may exhibit limited surface accessibility. Homogenizing the soil to <100 mesh minimizes textural heterogeneity, ensuring that any observed differences in desorption efficiency can be attributed primarily to the biosurfactant treatment rather than to variations in particle size distribution. Subsequently, 50 g of the prepared soil was placed into a 250 mL Erlenmeyer flask, followed by the addition of 50 mL of 1% biosurfactant solution (resulting in a soil-to-solution ratio of 1 : 1). The mixture was shaken at 200 rpm for 2 h, after which the supernatant was collected, and its crude oil content was determined to calculate the desorption efficiency. A parallel set of identical treatments was maintained for 2 days to investigate potential extended interactions or microbial responses under prolonged contact conditions.

### 2.6 16S rRNA and metagenomic analysis

To investigate the impact of biosurfactants on indigenous microbial communities, soil samples were collected before and after treatment. Total DNA was extracted from each sample using the QIAamp DNA Mini Kit (Qiagen) following the manufacturer's protocol. The extracted DNA samples were stored at −20 °C until further processing.

For taxonomic profiling, the 16S rRNA gene was amplified using primers targeting the V3–V4 region, with illumina adapter overhangs incorporated. PCR amplification was performed under the following conditions: initial denaturation at 95 °C for 30 s, followed by 30 cycles of denaturation at 95 °C for 30 s, annealing at 62 °C for 15 s, and extension at 72 °C for 45 s, with a final extension at 72 °C for 5 min. The illumina MiSeq platform using paired-end sequencing was used to sequence QC-qualified libraries. Raw sequencing data were processed using the QIIME toolkit (v1.8.0).^30^ Operational taxonomic units (OTUs) were clustered at a 97% similarity threshold against the Greengenes reference database.^31^

Taxonomic assignments was performed based on representative OTU sequences. For functional profiling, metagenomic sequencing data were analyzed by aligning annotated genes against the NCBI non-redundant (NR) database. Functional genes, modules, and pathways were annotated using the KEGG database.^32^

## 3. Results

### 3.1 Contaminant adsorption isotherms on different substrates

**Fig. 1:**
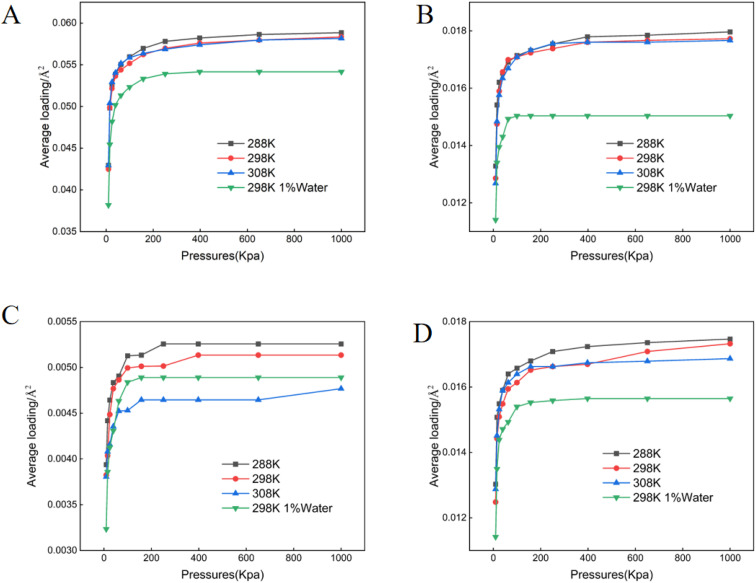
Adsorption isotherms of crude oil components on muscovite. (A) Aromatics (B) asphaltenes (C) resins (D) saturates.

**Fig. 2:**
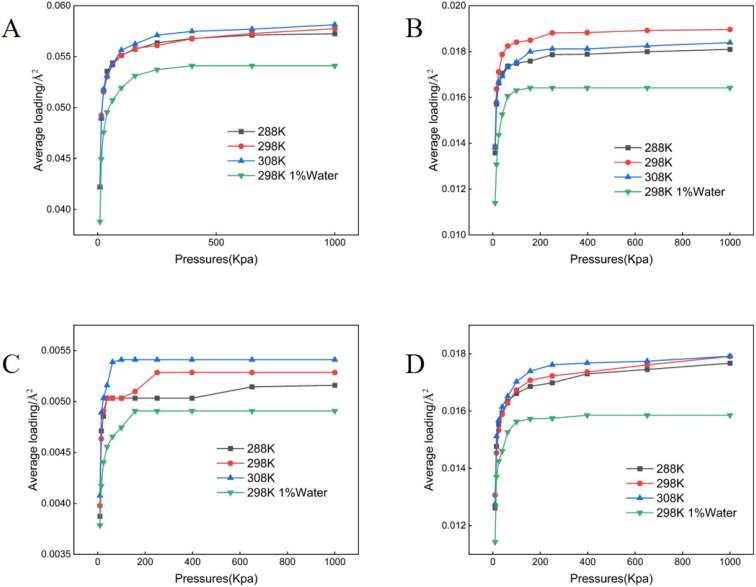
Adsorption isotherms of crude oil components on kaolinite. (A) Aromatics (B) asphaltenes (C) resins (D) saturates.

**Fig. 3:**
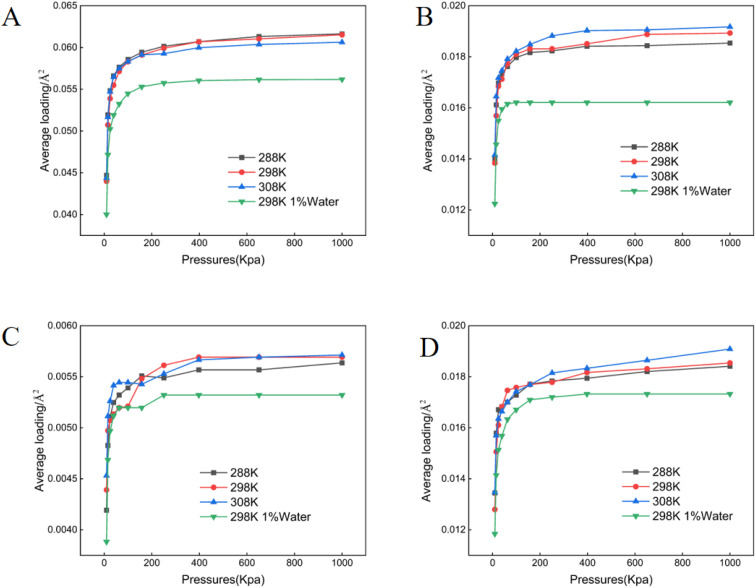
Adsorption isotherms of crude oil components on Ca^+^ montmorillonite. (A) Aromatics (B) asphaltenes (C) resins (D) saturates.

**Fig. 4:**
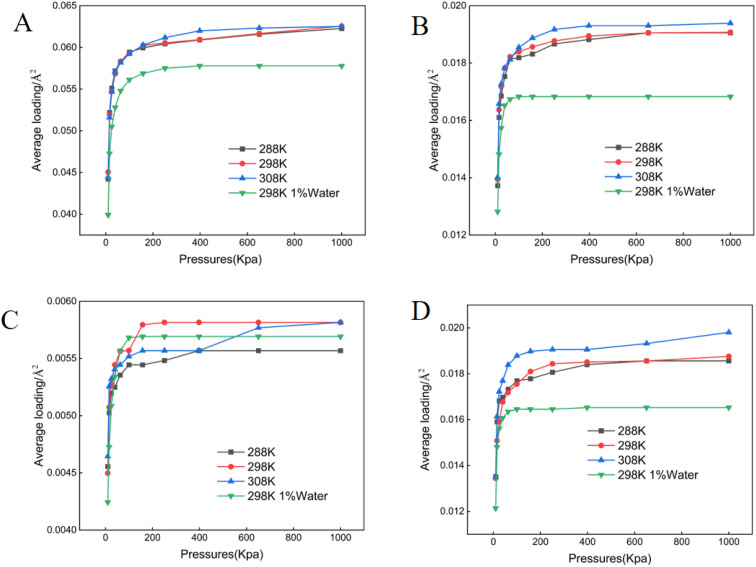
Adsorption isotherms of crude oil components on Na^+^ montmorillonite. (A) Aromatics (B) asphaltenes (C) resins (D) saturates.

**Fig. 5:**
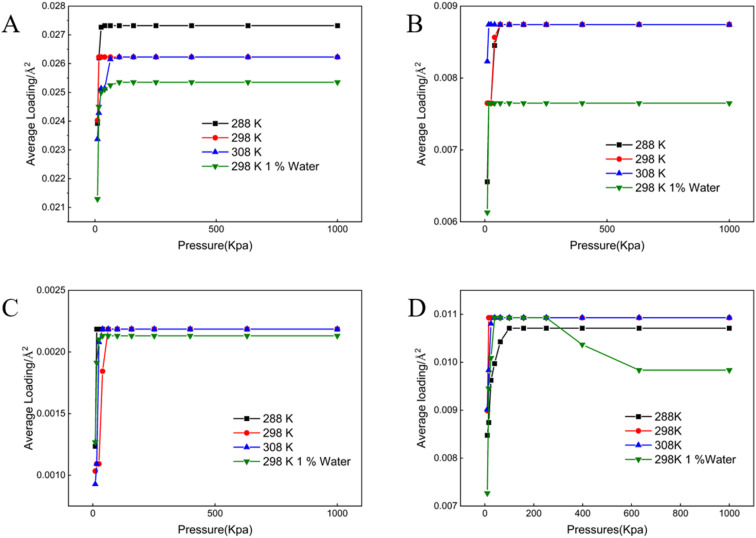
Adsorption isotherms of crude oil components on SIO_2_. (A) Aromatics (B) asphaltenes (C) resins (D) saturates.

All adsorption data generated in this study conformed to the Langmuir isotherm model (Fig. 1–5), indicating that each crude oil fraction—saturates, aromatics, resins, and asphaltenes attains a saturation point on the investigated minerals. Across all mineral substrates, aromatic compounds were consistently adsorbed most strongly, whereas resins exhibited the lowest adsorption capacity. Comparison of the mineral substrates revealed that SiO_2_ had a markedly lower adsorption capacity than kaolinite, muscovite, and montmorillonite. Specifically, for aromatics (the most strongly adsorbed fraction) SiO_2_ reached a maximum adsorption capacity of only 0.027 molecules per Å^2^, in contrast to 0.061–0.062 pcs per Å^2^ on Ca^2+^- and Na^+^-montmorillonite (Fig. 1–5). Kaolinite and muscovite displayed intermediate values of 0.057–0.058 pcs per Å^2^. These findings are consistent with earlier studies showing that layered silicate minerals such as montmorillonite can accommodate higher levels of hydrocarbons due to their larger specific surface area and greater cation exchange capacity (CEC).^33^

Interestingly, resins exhibited the most modest adsorption among the four crude oil fractions; their adsorption values on montmorillonite were only approximately 9.51% of those observed for aromatics (Fig. 3C and B). This finding suggests that the polar or colloidal fractions of heavy oil (*e.g.*, asphaltenes and resins) may not always dominate surface interactions; rather less polar but more structurally planar compounds (like aromatics) can achieve stronger substrate binding.^15^ A temperature increase within the range of 288–308 K resulted in a modest enhancement of adsorption for most fractions, in line with thermodynamic studies suggesting that increased kinetic energy can facilitate the movement of hydrocarbon molecules into mineral interlayers.^34^ Moreover, increasing in mineral moisture content significantly reduced adsorption capacity-particularly for asphaltenes and saturates. This effect likely arises from enhanced mineral hydrophilicity and the strong non-polarity of asphaltenes combined with saturate hydrocarbons, thereby diminishing hydrocarbon-substrate affinity.^12^

**Fig. 6:**
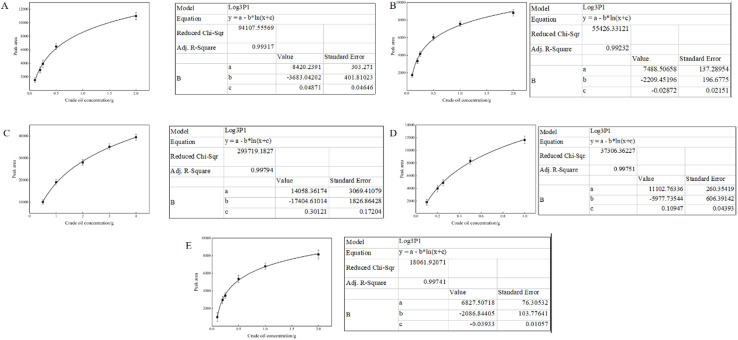
Adsorption curves of crude oil on different substrates. (A) Muscovite (B) Kaolinite (C) Ca^+^ montmorillonite (D) Na^+^ montmorillonite (E) SIO2.

Validation tests performed with real minerals similarly showed that all isotherms followed Langmuir behavior, corroborating the simulation results (Fig. 6). Montmorillonite (both Ca^2+^ and Na^+^ forms) exhibited the highest adsorption values (0.086–0.091 g g^−1^), whereas SiO_2_ consistently showed the lowest (0.013 g g^−1^). This congruence between the simulation results and experimental data indicated the reliability of the molecular dynamics approach for predicting crude oil behavior in heterogeneous soil matrices.

### 3.2 Desorption efficiency of different biosurfactants on pollutants

To screen for optimal desorbent candidates, we performed molecular dynamics simulations to compare the mean squared displacement (MSD) of pollutants, pollutant mineral interaction energies, and hydrogen-bond formation for four biosurfactants: sophorolipid (SL), trehalose lipid (TL), rhamnolipid (RL), and mannosylerythritol lipid (MEL). The presence of biosurfactants reduced the diffusion range of crude oil molecules compared to systems without surfactant, indicating a collective tendency to aggregate and emulsify crude oil components.

MSD values exhibited significant variability depending on the adsorption capacity of the substrate. SiO_2_, which had thelowest affinity to crude oil, showed the highest pollutant diffusion −1.35 to 10.98 times greater than that observed on other minerals. Among the biosurfactants tested, SL demonstrated exceptional performance on Ca^2+^-montmorillonite and kaolinite reducing MSD by 24.56% and 20.73%, respectively. In contrast, TL exhibited its strongest effect on SiO_2_ and Na^+^-montmorillonite, achieving reductions in pollutant diffusion of 30.94% and 20.89% respectively (Fig. S1).

Hydrogen bonding analysis aligned well with the MSD observations. SL and TL formed 1.76–8.47% more hydrogen bonds in the simulated systems than did RL or MEL. Higher levels of hydrogen bonding between biosurfactants and crude oil fractions can enhance micelle formation, thereby sequestering hydrocarbons away from mineral surfaces (Fig. S2).^35^ Additionally, interaction energy calculations consistently yielded negative values in the presence of all biosurfactants, indicating a strong tendency toward mineral–hydrocarbon dissociation. Notably, SL delivered the greatest enhancement in dissociation energy −15.52–113.97% higher than other biosurfactants—positioning SL as a promising universal candidate for crude oil desorption from soil surface (Fig. S3).

### 3.3 Bench-scale validation

We conducted bench-scale desorption experiments using natural minerals pre-saturated with crude oil in Fig. 6. Langmuir adsorption isotherms derived from these experiments consistently confirmed that montmorillonite exhibited the highest hydrocarbon loading (0.086–0.091 g g^−1^), whereas SiO_2_ showed the lowest (0.013 g g^−1^). When applied as a 1% solution, SL achieved the highest desorption efficiency across all tested minerals, ranging from 51.46% for SiO_2_ to 99.63% for Na^+^-montmorillonite. The second-best overall performance was observed with MEL and RL, while TL surprisingly exhibited lower efficiency, potentially due to its stronger hydrophobicity and relatively limited solubility under aqueous conditions. These results demonstrate a key limitation of certain biosurfactants: although TL showed promise in simulation for specific substrates, it did not disperse effectively in real aqueous systems. This finding highlights the importance of validating computational predictions with empirical tests.^36^

A slight discrepancy was noted between simulations and bench-scale experiments for muscovite and kaolinite, with muscovite exhibiting significantly lower desorption than predicted from the simulations. This divergence may arise from the infiltration of water molecules into the mineral lattice under real conditions, altering the effective surface area and surface energy in ways not fully captured in the simulations.^37^ Nonetheless, the overall trends were highly consistent, reinforcing the robustness of the molecular dynamics framework for screening potential desorbents.

### 3.4 Desorption experiments on field soil samples

To assess practical applicability, desorption experiments were conducted using the actual oil-contaminated soil, XRD analysis revealed that the soil was primarily composed of SiO_2_ (51.8%) and kaolinite (25.6%), with minor fractions of plagioclase, potassium feldspar, illite, calcite, and hematite (each <10%) (Fig. S4). The soil was characterized as sandy loam with a crude oil content of 0.8% and a moisture content of 35.2%. Based on the simulation and bench-scale experimental results, SL was selected as the most promising desorbent for further evaluation.

#### 3.4.1 Simulated mineral blends *vs.* actual soil

**Fig. 7:**
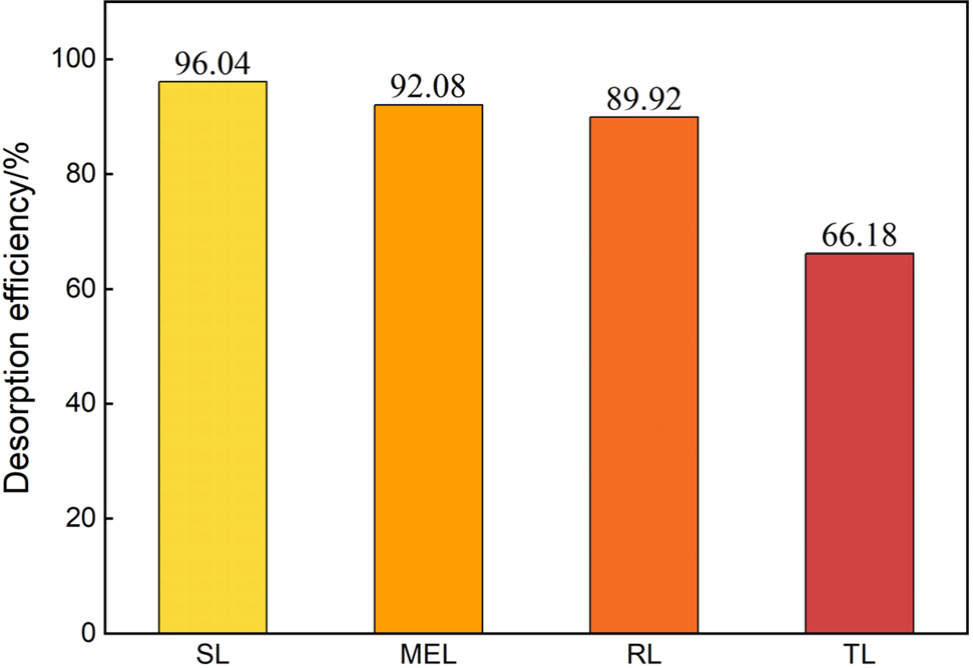
Desorption efficiency of on-site soil samples.

Synthetic mineral mixtures designed to emulate the soil composition (51.8% SiO_2_ and 25.6% kaolinite.) exhibited SL desorption efficiencies of 65.37% when fully saturated with crude oil and 61.69% at contaminant levels simulating field conditions, indicating a negative correlation between total oil loading and desorption efficacy. Subsequent trials on field-collected samples (Fig. 7) showed that SL removed 96.04% of residual crude oil, followed by MEL (92.08%), RL (89.92%), and TL (66.18%).

### 3.5 Soil microbial community analysis

**Fig. 8:**
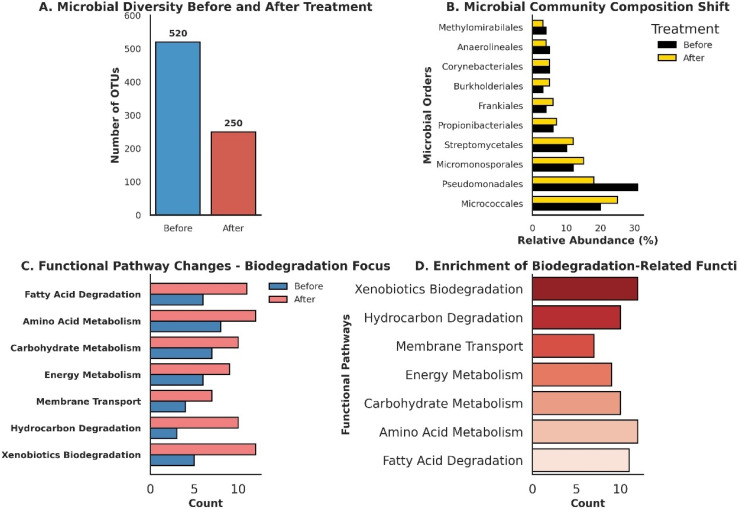
Microbial community and metabolism shift before and after biosurfactant adding as desorbent. (A) A significant reduction in microbial diversity (OTU counts); (B) changes in microbial composition, with increased abundance of hydrocarbon-degrading taxa; (C) a comparison of functional pathway counts; (D) enrichment of key biodegradation-related functions after treatment.

The dual role of biosurfactants in enhancing hydrocarbon desorption and modulating the structure and function of soil microbial community was evaluated. Fig. 8A shows a significant reduction in the number of operational taxonomic units (OTUs) following biosurfactant treatment, suggesting a shift in microbial diversity driven by selective pressure favoring hydrocarbon-degrading taxa. This observation is consistent with previous studies that indicate biosurfactants can act as bioavailability enhancers, leading to the proliferation of specialized microbial consortia capable of utilizing hydrocarbons as carbon sources.^36,38^ Analysis of the relative abundance of dominant bacterial orders revealed a marked decline in *Pseudomonadales* and a pronounced increase in *Micromonosporales* and *Streptomycetales*, —both of which are known to harbor potent hydrocarbon degraders (Fig. 8A and B). These shifts were in line with findings from Mandal, who reported that biosurfactant application fosters microbial succession by promoting taxa with enhanced biodegradation capabilities.

Fig. 8C and D illustrate functional pathway shifts in response to biosurfactant treatment, as determined by KEGG annotation. Post-treatment microbial communities exhibited a substantial increase in pathways associated with xenobiotics biodegradation and metabolism, amino acid metabolism, and membrane transport, suggesting physiological adaptation toward hydrocarbon degradation. These results support prior research indicating that biosurfactants can trigger metabolic reprogramming in soil microbiomes, leading to an upregulation of hydrocarbon catabolic genes.^39^ Moreover, the increased abundance of pathways related to fatty acid metabolism suggests that biosurfactants not only enhance hydrocarbon desorption but also facilitate microbial uptake and subsequent oxidation of released hydrophobic compounds. In contrast, pathways associated with digestive system functions and environmental adaptation were relatively suppressed, indicating that the microbial community is shifting toward a niche optimized for hydrocarbon degradation rather than generalist survival strategies.

These results highlight the efficiency of biosurfactants not only as desorbents but also as bioaugmentation enhancers, reinforcing the concept that chemical and biological remediation strategies can act synergistically. The observed microbial shifts are consistent with previous studies demonstrating how biosurfactants can both disrupt hydrocarbon–mineral interactions and create a more favorable environment for pollutant-degrading microbial populations.^34,40^ Such findings underscore the potential of biosurfactant-mediated soil remediation as a sustainable alternative to conventional chemical treatments, minimizing secondary pollution risks while leveraging natural attenuation mechanisms. Further investigations integrating metagenomic and transcriptomic analyses could provide deeper insights into the genetic mechanisms underlying these community shifts and help optimize biosurfactant formulations for site-specific remediation challenges.

## 4. Discussion

This study systematically investigated the interplay among soil mineral composition, crude oil components, and biosurfactant desorbents, thereby providing mechanistic insights into the remediation of heavy oil – contaminated soils. Our molecular dynamics simulations, supported by bench-scale and field experiments, revealed several critical factors governing adsorption and desorption processes: (i) the structural and chemical properties of soil minerals; (ii) the compositional variability among crude oil fractions (*e.g.*, aromatics, resins, asphaltenes, and saturates); (iii) the physicochemical attributes of the biosurfactants employed, and (iv) the biochemical function of biosurfactants of microbial activity.

### 4.1 Influence of mineral composition on adsorption

Mineral composition emerged as a key determinant of crude oil adsorption, in agreement with prior reports that highlight the importance of mineral lattice structure, cation exchange capacity (CEC), and specific surface area.^12,13^ Our findings demonstrated that montmorillonite (both Ca^2+^- and Na^+^-enriched) exhibited the highest adsorption capacities for crude oil fractions (Fig. 1–5), a result consistent with its well-known 2 : 1 layered structure and robust swelling behavior.^14^ Specifically, we observed that montmorillonite adsorbed aromatic constituents at levels exceeding 200% of those on SiO_2_, paralleling other studies which have linked montmorillonite's large interlayer spacing and high CEC to enhanced pollutant uptake.^41^ Conversely, non-expanding minerals such as kaolinite—composed of alternating silica tetrahedral and alumina octahedral layers, showed moderately lower adsorption. This trend is supported by prior work indicating that kaolinite's strong hydrogen bonding between layers suppresses interlayer expansion, limiting available adsorption sites.^42,43^ SiO_2_, with a lower surface charge and simpler structure, exhibited the lowest crude oil adsorption, thus reaffirming the notion that structure complexity and charge distribution significantly influence sorption behavior.^44^

### 4.2 Mechanisms underlying biosurfactant efficiency

Our simulations further revealed that biosurfactant-mediated reduction of oil–mineral interaction energies can substantially promote pollutant desorption. Among the applied biosurfactants, sophorolipid (SL) consistently exhibited the highest efficiency across mineral substrates, exceeding alternative surfactants by 15.52–113.97% in terms of crude oil dissociation (Fig. 6 and 7). These observations are consistent with hypotheses suggesting that biosurfactants reduce interfacial tension and alter surface wettability.^40,45^ SL's amphiphilic structure, along with its relatively balanced hydrophobic–hydrophilic profile, may facilitate adsorption at the oil–water interface and enhance solubilization of hydrophobic fractions.^39^ Trehalose lipids (TL) also demonstrated promising results in specific scenarios (*e.g.*, Na^+^-montmorillonite or high-SiO_2_ content soil), likely due to favorable hydrophobic interactions that partition biosurfactant molecules to the oil phase.^46^ However, the relatively poor desorption performance of TL in bench-scale aqueous systems highlights the complexity of biosurfactant–soil interactions, where factors such as local mineral hydrophilicity, aqueous solubility, and micelle formation all modulate remediation outcomes.

### 4.3 Contextualizing adsorption–desorption dynamics

The adsorption–desorption trends observed in this study align with and extend prior research examining how water content, mineralogy, and pollutant chemistry synergistically affect remediation efficiency.^34,47^ Our data confirmed that increasing soil moisture content reduces crude oil adsorption and enhances biosurfactant-mediated treatment, possibly by increasing soil porosity and decreasing soil cohesion.^48,49^ Notably, aromatics, which exhibited the highest mineral adsorption, also demonstrated relatively high rates of desorption upon biosurfactant treatment, underscoring the need for tailored remediation approaches that address both the polar and non-polar fractions of crude oil.^15^

### 4.4 Implications for soil ecology and sustainability

Beyond pollutant removal, our field-scale experiments revealed notable shifts in microbial community composition—particularly an increase in Gram-positive actinomycetes after treatment, which can accelerate further biodegradation of residual oil.^38^ While overall microbial abundance decreased, likely due to initial biosurfactant toxicity or nutrient depletion, the emergence of actinomycetes and other aerobic bacteria could offer long-term benefits for soil recovery and fertility.^36^ Future work should examine co-remediation strategies that couple biosurfactant washing with biostimulation, ensuring not only high immediate contaminant removal but also sustained microbial degradation.

The proposed synergistic effect between biosurfactant-induced desorption and biodegradation is inherently concentration-dependent. At an optimal concentration (*e.g.*, the 1% w/v used in this study), biosurfactants effectively solubilize heavy oil without exerting significant toxicity on the indigenous microbial community, thereby enhancing hydrocarbon bioavailability.^50,51^ This is consistent with findings that biosurfactants at appropriate concentrations (*e.g.*, 1 × CMC) significantly improve contaminant removal efficiency by increasing mass transfer and bioavailability. However, excessively high biosurfactant concentrations could potentially inhibit microbial activity due to membrane disruption, increased cellular stress, or preferential utilization of the biosurfactant as a carbon source over the target contaminants. Akbari *et al.*^52^ demonstrated that high-dose rhamnolipid increased bacterial population size but paradoxically hindered hydrocarbon biodegradation (23% *vs.* 40% at lower doses), highlighting the inhibitory effects of excessive biosurfactant application. Similarly, Gill *et al.*^53^ reported that surfactant exposure led to the loss of up to 36% of microbial OTUs, indicating significant community restructuring at higher concentrations. While our study focuses on demonstrating this synergy at a single, effective concentration, the quantitative relationship between biosurfactant dose, desorption efficiency, and microbial stimulation warrants dedicated future investigation to define the optimal operational window for field applications. As demonstrated by Shah *et al.*^54^ for sophorolipids in phytoremediation systems, optimal concentrations (*e.g.*, 0.25 g kg^−1^) exist beyond which remediation efficacy declines, underscoring the need for dose optimization in large-scale applications.

### 4.5 Other possible influencing factors

The remediation of crude oil-contaminated soils using biosurfactants is a multifaceted process influenced by soil physicochemical properties, mineralogical characteristics, and environmental conditions. Soil pH plays a pivotal role in modulating the surface charge of minerals and the ionization state of biosurfactants, thereby affecting adsorption–desorption equilibria. For instance, under acidic conditions (pH < 5), the protonation of hydroxyl groups on clay minerals like montmorillonite enhances their affinity for non-polar hydrocarbons, while alkaline conditions (pH > 8) promote deprotonation, increasing surface hydrophilicity and favoring desorption.^12^ Biosurfactants such as sophorolipid exhibit pH-dependent micellization, with optimal emulsification efficiency near neutral pH.^39^ Deviations from this range can destabilize micelles, reducing hydrocarbon solubilization and desorption efficacy.

Ionic strength also significantly impacts biosurfactant performance by altering interfacial interactions. High ionic strength compresses the electrical double layer, reducing repulsion between negatively charged biosurfactant molecules and mineral surfaces, thereby enhancing adsorption.^10^ However, excessive salinity may precipitate biosurfactants or elevate critical micelle concentrations (CMCs), as demonstrated for trehalose lipid in saline environments.^26^ Field soils with heterogeneous ion compositions further complicate remediation, as divalent cations like Ca^2+^ promote clay aggregation, reducing pore accessibility and hydrocarbon mobility.^34^

Although molecular simulations offer valuable mechanistic insights, certain complexities (such as localized mineral defects, natural organic matter, and *in situ* heterogeneity) cannot be fully captured *in silico*.^11^ Additionally, parameters like pH, salinity, and surfactant biodegradability in field soils warrant deeper investigation to optimize biosurfactant use.^10^ Integrating advanced *in situ* characterization techniques with multi-scale modeling may help refine predictions and bridge bench-scale findings to full-scale applications. Further exploration of biosurfactant, for example, mineral synergy through engineered modifications of clay surfaces or specialized biosurfactant formulations, could pave the way for cost-effective and eco-friendly remediation solutions in large-scale oilfield operations.

While this study provides valuable insights into the mechanisms of biosurfactant-driven desorption and biodegradation of heavy oil-contaminated soils, several limitations should be acknowledged. Firstly, the experimental conditions, such as controlled temperature and moisture levels, may not fully replicate the dynamic and heterogeneous nature of actual contaminated sites. For instance, the influence of variable soil pore structures and groundwater flow on biosurfactant distribution and microbial activity remains unexplored. Secondly, the study focused on short-term desorption efficiency (up to 2 days), lacking long-term assessments of biosurfactant persistence and potential ecotoxicity. Chronic exposure to biosurfactants could alter soil microbial community dynamics or lead to the development of resistant microbial strains, as observed in similar systems. Thirdly, the mineralogical complexity of real soils, which often contain mixed clay minerals like illite and chlorite alongside variable organic matter content, was simplified in this study. Future work should incorporate more representative soil matrices to enhance applicability. Lastly, while sophorolipid demonstrated superior performance in this study, its efficacy across diverse crude oil types (*e.g.*, varying asphaltene/resin ratios) and environmental conditions (*e.g.*, saline soils) warrants further investigation. Addressing these gaps will strengthen the translational potential of biosurfactant-based remediation strategies.

In summary, this study underscores the critical importance of mineral composition, crude oil fraction characteristics, and biosurfactant chemistry in shaping the adsorption–desorption dynamics of contaminated soils. By coupling molecular simulations with empirical validation, we demonstrate that sophorolipid-based formulations can deliver high desorption efficiencies across diverse substrates, thereby providing a promising avenue for green and sustainable remediation in oil-contaminated environments.

## 5. Conclusion

This study systematically elucidates the multifaceted interrelationships among three core factors—soil mineral composition, crude oil fraction characteristics, and biosurfactant physicochemical properties—in governing the efficacy of oil-contaminated soil remediation strategies. Through integrated molecular dynamics simulations and bench-scale validation experiments, it was revealed that soil minerals with high cation exchange capacity (CEC), notably montmorillonite, exhibit enhanced adsorptive capacity for heavy oil fractions, primarily attributed to their expanded surface area and electrostatic interactions with hydrophobic contaminants. Concurrently, sophorolipid biosurfactants demonstrated superior performance across diverse crude oil matrices, outperforming other tested surfactants in desorbing even the most strongly adsorbed aromatic hydrocarbons, a critical advantage for targeting recalcitrant contaminants. Beyond direct pollutant mobilization, the study uncovered a synergistic effect: biosurfactant application simultaneously stimulated indigenous hydrocarbon-degrading microbial communities, as evidenced by increased abundance of functional genes and enhanced biodegradation rates. This dual mechanism—physical contaminant solubilization coupled with biological degradation—underscores the holistic value of biosurfactant-enhanced remediation: not only improving short-term pollutant removal efficiency but also fostering long-term *in situ* bioremediation potential. Looking forward, expanded field-scale investigations under variable environmental conditions are needed to validate lab-scale findings, alongside the development of site-specific biosurfactant formulations tailored to local mineralogy and contaminant composition. Collectively, these insights bridge computational modeling with practical application, advancing the development of sustainable, science-driven solutions for the bioremediation of crude oil-contaminated soils.

## Author contributions

Qi Xiu: writing – original draft, investigation, validation; Honglin He: validation; Zhenghui Liu: data curation, validation; Xuan Ou: data curation, sampling; Yifei Meng, Kangbo Zhao, Qian Yang, Yahan Hou & Xinrui Zhang: validation; Shun Yao: data curation, writing; Peike Gao: data curation, writing; Wenjie Xia: conceptualization, investigation, writing – original draft, methodology, data curation, validation, supervision, funding acquisition.

## Conflicts of interest

The authors declare that they have no known competing financial interests or personal relationships that could have appeared to influence the work reported in this paper.

## Supplementary Material

RA-016-D5RA09479H-s001

## Data Availability

All data generated or analysed during this study are included in this published article. Supplementary information (SI) is available. See DOI: https://doi.org/10.1039/d5ra09479h.
