# Supplementary material for: Biosurfactant-driven desorption and remediation of heavy oil contaminated soils underpinned by molecular simulations and microbial dynamics
Source: RSC Adv. 2026 Mar 25;16(18):16316–28. doi: 10.1039/d5ra09479h (PMC13014477; doi:10.1039/d5ra09479h)
Supplement: RA-016-D5RA09479H-s001 [file RA-016-D5RA09479H-s001.pdf]

Supplementary Information (SI) for RSC Advances.
This journal is © The Royal Society of Chemistry 2026

Supplementary Information for

Biosurfactant-Driven Desorption and Remediation of Heavy Oil Contaminated Soils Underpinned by Molecular Simulations and Microbial Dynamics

Qi Xiu [a,1], Honglin He [a,1], Zhenghui Liu [b,1], Xuan Ou [a], Yifei Meng [a], Kangbo Zhao [a], Qian Yang [a], Xingrui Zhang [a], Yahan Hou [a], Shun Yao [a,*], Peike Gao [c,*], Wenjie Xia [a,*,1]

* E-mail: Wenjie. Xia (wenjie.xia@nankai.edu.cn)

The file includes:

Supplementary FIGURES (1-5)

Supplemental Figures

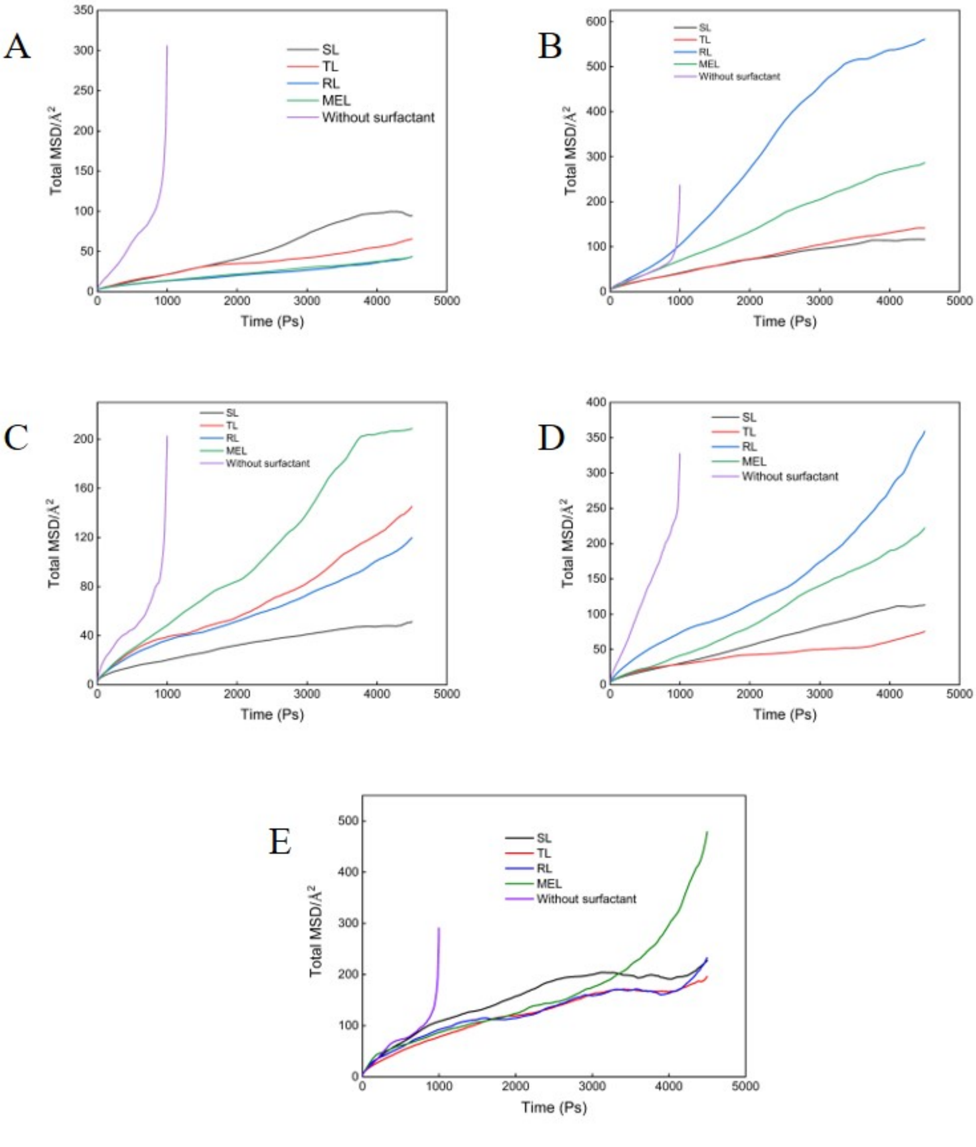

Fig S1. MSD of crude oil on different substrate. (A) Muscovite (B) Kaolinite (C) Ca^{+} montmorillonite (D) Na^{+} montmorillonite (E) SIO_2

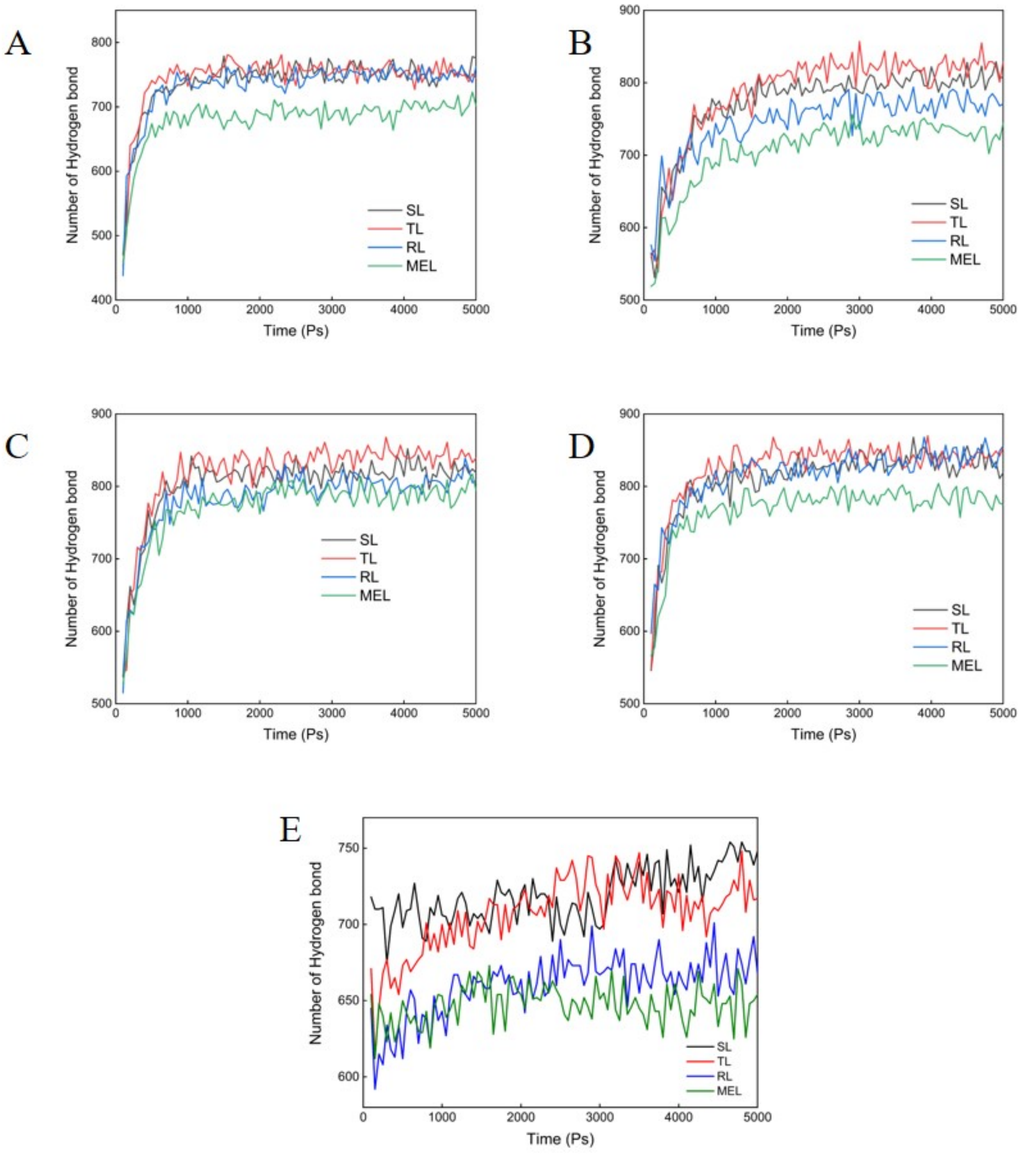

Fig S2. Number of hydrogen bond of crude oil on different substrates. (A) Muscovite (B) Kaolinite (C) Ca^+ montmorillonite (D) Na^+ montmorillonite (E) SIO_2

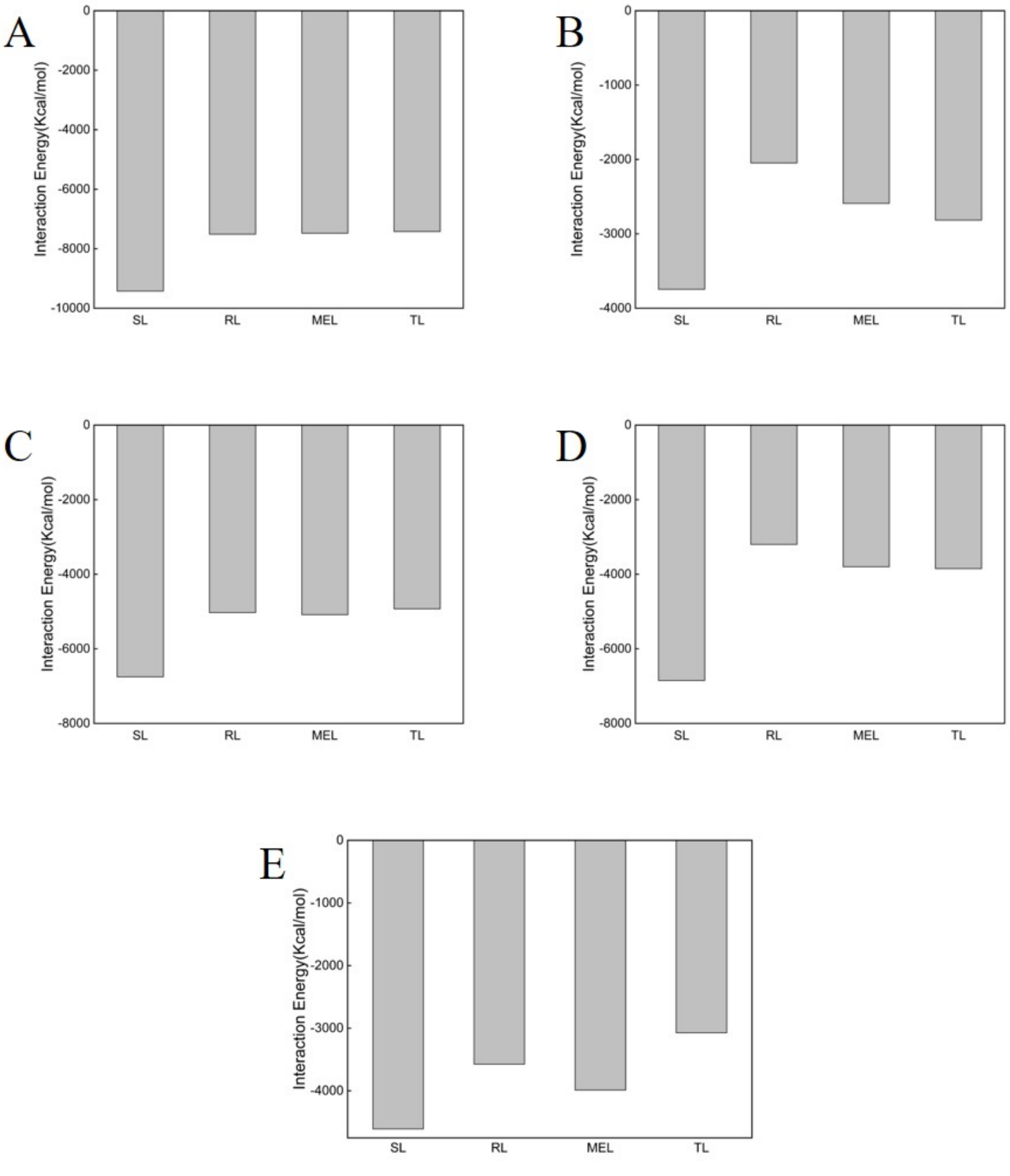

Fig S3. Interaction energy between crude oil and substrate. (A) Muscovite (B) Kaolinite (C) Ca^{+} montmorillonite (D) Na^{+} montmorillonite (E) SIO2

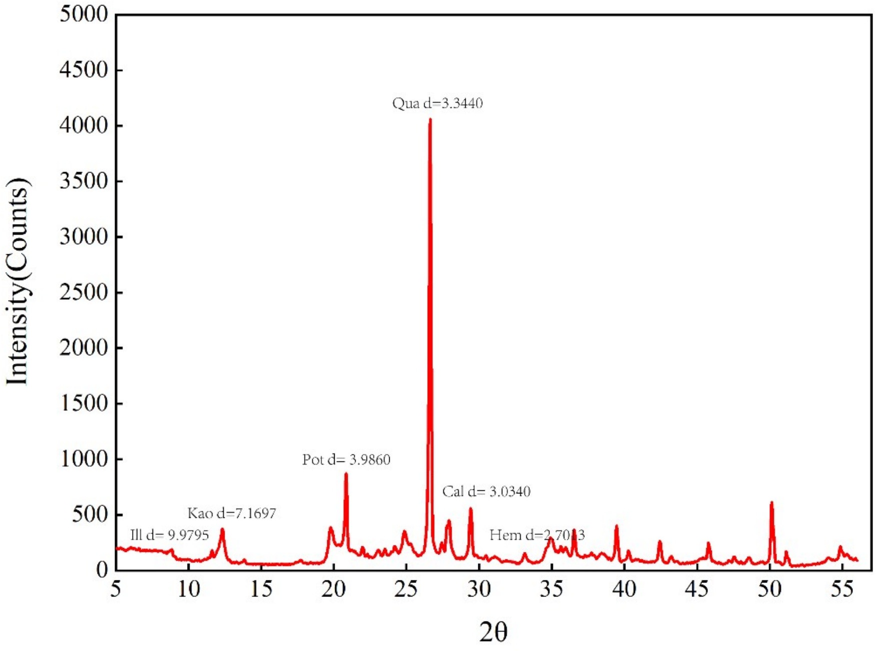

Fig S4. Mineral composition analysis of soil samples from Jilin oilfield

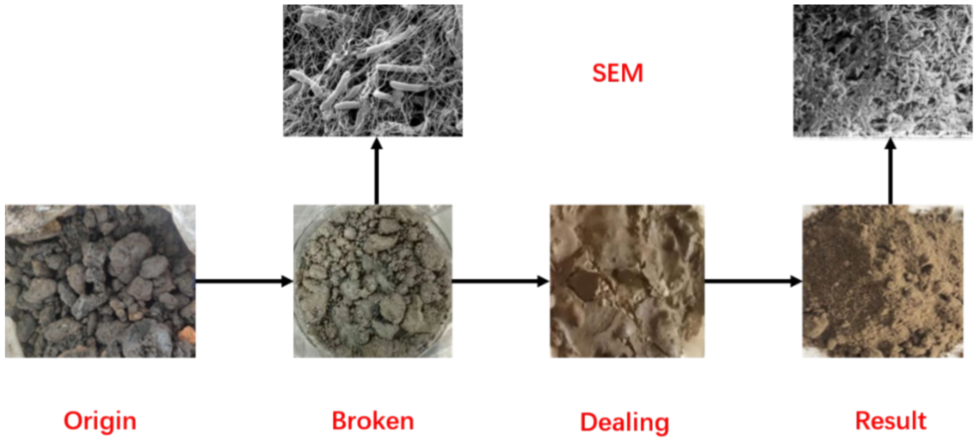

Fig S5. Processing of on-site samples for subsequent desorption experiments
